# Two cases of giant mucinous cystadenomas in postmenopausal women

**DOI:** 10.1093/jscr/rjae833

**Published:** 2025-01-03

**Authors:** Shumarova Svetlana, Anatoli Semkov, Vesela Karamisheva

**Affiliations:** Department of Surgery, University Hospital “Aleksandrovska”, B-1431 Sofia, Bulgaria; Medical University, Sofia, Bulgaria; Medical University, Sofia, Bulgaria; Department of Thoracic Surgery, UMHAT St Ivan Rilski, Sofia, Bulgaria; Faculty of Medicine, Department of Obstetrics and Gynecology, Medical University of Sofia, Sofia, Bulgaria; SBALAG “Maichin dom”, Sofia, Bulgaria

**Keywords:** ovarian cyst, mucinous cyst, ovarian tumor, serous ovarian cyst

## Abstract

Mucinous ovarian cystadenomas are rare epithelial benign tumors that can reach significant sizes. They are often asymptomatic and are discovered incidentally during an ultrasound examination. We present two clinical cases of mucinous ovarian cystadenomas with abdominal distention. Complicated variants can mimic an acute surgical abdomen and often require the involvement of a multidisciplinary team in operative treatment. Timely diagnosis and treatment are of utmost importance to prevent complications and malignancy.

## Introduction

Ovarian cystadenomas are benign neoplasms, with serous and mucinous variants being the most common [1]. Serous cystadenomas do not have mutations in either KRAS or BRAF, whereas mucinous cystadenomas have KRAS mutations in up to 58% [1]. Ovarian cystadenomas are very often discovered accidentally, without specific clinical symptoms, and in complicated forms with the presence of abdominal pain, it can imitate an acute surgical abdomen and require joint team treatment by a gynecologist and a surgeon. Untreated, they can grow to significant sizes and cause complications such as abdominal compartment syndrome [2], torsion [3, 4], and rupture [5], which require immediate surgical intervention. We describe two clinical cases of large mucinous ovarian cysts, one of which was in combination with intramural uterine leiomyoma.

## Case 1

**Figure 1:**
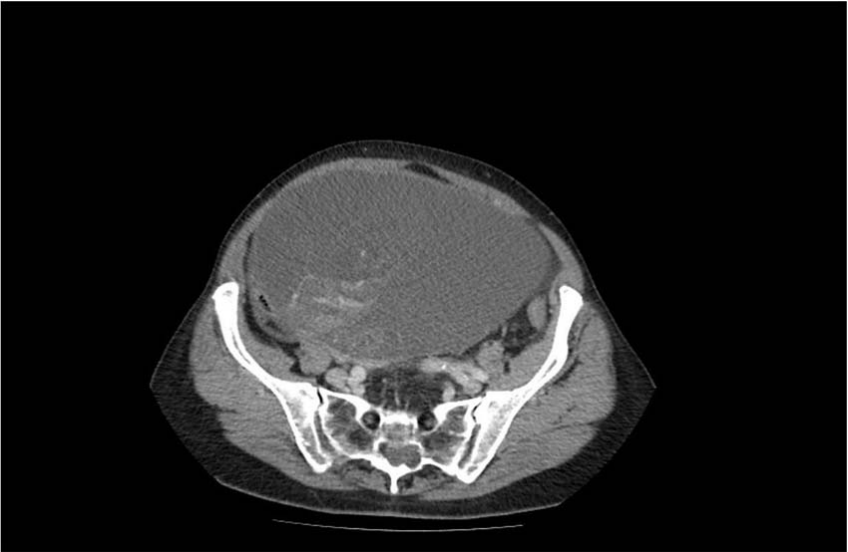
CT image of a large ovarian mucinous cystadenoma of the left ovary.

**Figure 2:**
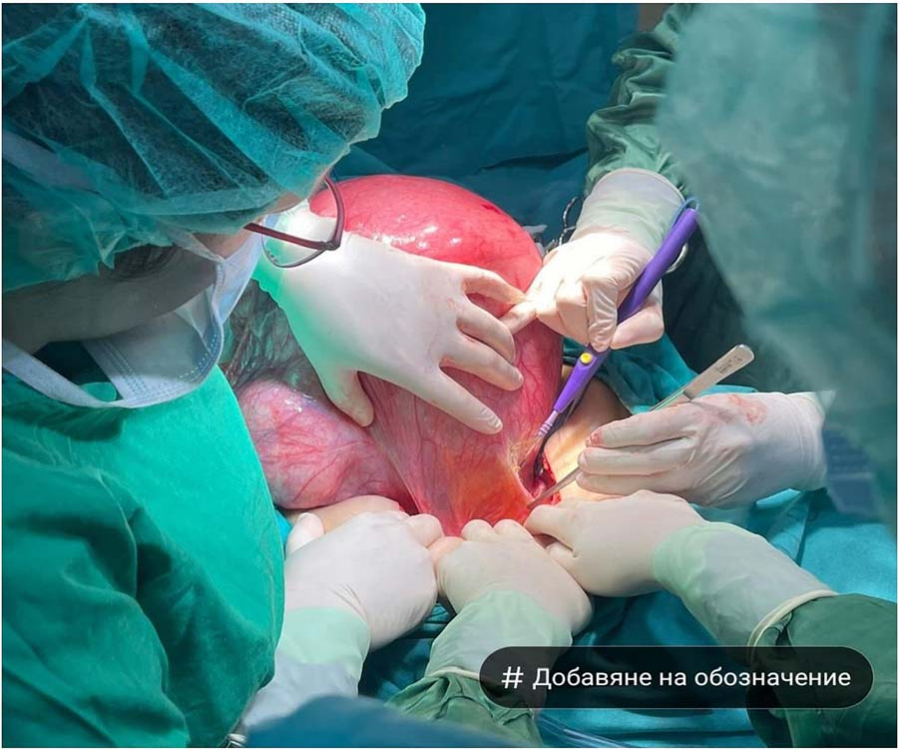
Macroscopic view of a large cystadenoma of the left ovary—lower median laparotomy.

A 75-year-old woman with a complaint of heaviness in the abdomen with gradually increasing dimensions, as well as heaviness in the groin areas with pronounced constipation for several years, was consulted by a surgeon. Abdominal ultrasound revealed a large cystic formation with a hypoechoic appearance with isoechoic components in it with size of ~198 mm/d, compressing adjacent distended intestinal loops. To the left of it, a smaller structured cystic zone ~5 cm/d was visualized. Subsequent computed tomography (CT) of the abdomen and pelvis with contrast revealed a well-demarcated large cystic mass on the right of the pelvis, tracing cranially preaortic, retroperitoneally and reaching the level of the epigastrium, measuring 150/210/138 mm/d, thin-walled with homogenous contents and an adherent appendix to the wall. Numerous thin septa were visualized inside (Fig. 1). A second finding with a cystic structure is present on the left of the small pelvis with a size of 69/85/100 mm/d in contact with the main formation along the lower lateral and a contour of 7 cm/d. There is a herniation of the right lateral surface of the bladder into the right inguinal canal, a transitional herniation of the sigmoid to the left inguinal canal. Computed tomography confirm an ovarian cystadenoma on the right. Laboratory indicators did not give a deviation. A joint decision was made with a gynecologist to perform a laparotomy with hysterectomy (Fig. 2), bilateral adnexectomy, and appendectomy. Intraoperatively, the finding was consistent with that described by CT. The histology result revealed a serous ovarian cystadenoma on the left and fallopian tube with an abudance of cystically transformed Walthard’cell nests. The same changes were also described in the right fallopian tube, mucinous ovarian tumor with borderline malignancy, and appendix fibrinous purulent periappendicitis. The patient had no postoperative complications and was discharges on the seventh postoperative day.

## Case 2

**Figure 3:**
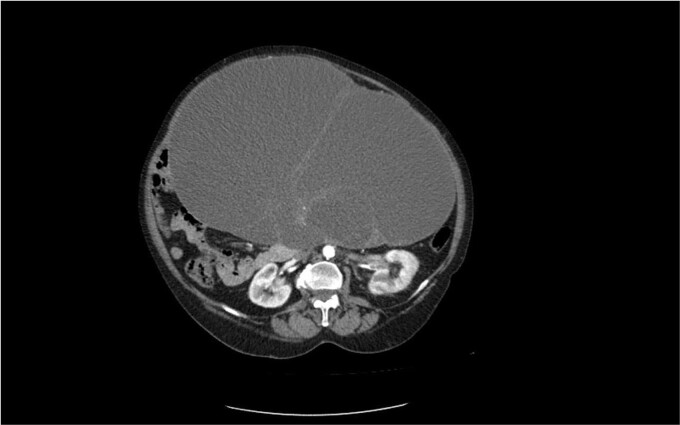
CT image of a mucinous cystadenoma of the left ovary.

The second case is of a 62-year-old woman, admitted to a surgery clinic with abdominal distention, difficult defecation, and ultrasound data of a large tumor in the abdomen. CT describes a large tumor formation, reaching ventrally to the anterior abdominal wall, measuring 317/202/272 mm/d with a predominantly cystic structure (Fig. 3). The finding has a homogeneous content and multiple intralesional septa with a mass effect in relation to small intestine, main abdominal vessels, compress and dislocates the transvers colon and stomach. Operative treatment followed with laparotomy and evacuation of about 8 l of serous fluid from the cystic formation described on CT. Given the concerns about the presence of malignancy, a hysterectomy with adnexectomy and appendectomy was performed. Histopathologically, a multilocular mucinous cystadenoma of the left ovary was proven. The uterus has cystic endometrium and myometrium with intramural leiomyomas and adenomyosis, and the appendix with fibrinous-purulent periappendicitis. The patient recovered and was discharged on the seventh postoperative day without complications.

## Discussion

Mucinous cystadenomas are more common—about 80% of the ovarian mucinous tumors [1] occurring most often in the third to sixth decade. Serous cystadenomas occur more often between the ages of 40 and 60, accounting for 16% of all ovarian epithelial neoplasms [1]. Very often the complaints are nonspecific such as abdominal distention and pain, and less often nausea, vomiting, and difficulty breathing. They can be asymptomatic with smaller cyst sizes and be found incidentally on an ultrasound examination. They are usually described as unilocular masses with a flat surface, anechoic, sometimes containing sonographically detectable septations. The most reliable methods for visualizing the cysts and establishing their origin are CT and magnetic resonance imaging.

Regarding some tumor markers that could be associated with a certain ovarian disease, there is still no consensus on their role in the differentiation between different types of benign ovarian tumors. Some authors found normal values of CA 125 in ovarian cystadenomas [3, 4], but others reported elevated values of CA 125 and CA 19-9 [5]. A recent report analyzed tumor markers in the differential diagnosis of benign ovarian masses [6]. A total of 135 patients were divided into four groups: Group A had simple luteal cyst (*n* = 18, 1.3%), group B had ovarian mature cystic teratoma (OMCT)(*n* = 57,42.2%), group C had ovarian endometriosis (*n* = 32;23.7%), and group D had ovarian epithelial tumors, including mucinous cystadenoma and serous cystadenoma (*n* = 28,20.8%). The results of the analysis suggest that CA 125 levels in endometriosis and SCC levels in OMCT may have a certain diagnostic value [6]. Receiver operating characteristic (ROC) analysis revealed that the combination of CA 125 and CA 19-9 had a higher diagnostic rate for benign epithelial tumors (AUC = 0.792; sensitivity: 64.5%; specificity: 85.7%).

Surgical treatment is tailored to the patient’s age, and in postmenopausal women or suspected malignancy, total hysterectomy with bilateral salpingo-oophorectomy is preferred. Although cystadenomas are benign epithelial tumors, cases of a combination of cystadenomas with anaplastic carcinoma [7–9] and malignant ovarian Brenner tumor [10] have also been reported. There is no definite algorithm for the volume of operative resection. We cannot assert that malignancy is to be expected primarily in postemenopausal women, since of the cases cited in one the woman was of reproductive age. Management of ovarian cystadenomas generally depends on a number of factors, such as symptoms, cyst size, age, medical history, and menopausal state of the patient [11].

## Conclusion

Ovarian cyst are rare in postmenopausal women, but their combination with other tumors can lead to a risk of malignant transformation. Therefore, they should be thoroughly investigated and exclude a malignant component that may be relevant to the patient’s prognosis.
